# Advances in Microbial Diagnostics: Machine Learning and Nanotechnology for Zoonotic Disease Control

**DOI:** 10.1002/wnan.70050

**Published:** 2026-01-22

**Authors:** Narges Lotfalizadeh, Cinzia Santucciu, Valentina Chisu, Helia Sepahvand, Abbas Rahdar, Razieh Behzadmehr, Octavio Luiz Franco, Guettari Moez, Luiz Fernando Romanholo Ferreira

**Affiliations:** ^1^ Department of Clinical Sciences, Faculty of Veterinary Medicine Shiraz University Shiraz Iran; ^2^ WOAH and NRL for Echinococcosis, Animal Health Istituto Zooprofilattico Sperimentale della Sardegna Sassari Italy; ^3^ Veterinary Medicine, Tehran University Tehran Iran; ^4^ Department of Physics University of Zabol Zabol Iran; ^5^ Department of Radiology Zabol University of Medical Science Zabol Iran; ^6^ Centro de Análises Proteômicas e Bioquímica (CAPB), Programa de Pós‐Graduação Em Ciências Genômicas e Biotecnologia Universidade Católica de Brasília (UCB) Brasília Brazil; ^7^ S‐Inova Biotech, Programa de Pós‐Graduação Em Biotecnologia Universidade Católica Dom Bosco (UCDB) Campo Grande Brazil; ^8^ Département de Physique et STI Tunis Guettari Moez Tunis University Tunis Tunisia; ^9^ Graduate Program in Genomic Sciences and Biotechnology Catholic University of Brasília Brasília Brazil

**Keywords:** biosensors, machine learning, microbial diagnostics, nanotechnology, zoonotic pathogens

## Abstract

Zoonotic diseases pose significant global health threats, with microbial pathogens, including bacteria, viruses, fungi, and protozoa, responsible for severe outbreaks. The rapid identification and control of zoonotic pathogens remain a major challenge due to their complex transmission dynamics and environmental persistence. Recent advances in molecular microbiology, nanotechnology, and artificial intelligence (AI) have revolutionized diagnostic and therapeutic strategies, enhancing the detection, monitoring, and prevention of diseases caused by pathogens. In machine learning (ML), it is possible to predict outbreaks and classify pathogens with high precision using genomic, proteomics, and epidemiological data, which can be analyzed with machine learning methods. Molecular‐level detection is possible with nanotechnology‐based biosensors, enabling rapid diagnosis even in areas with limited resources. Machine learning‐driven computational models and nanotechnology‐based detection tools can drive further advancements in microbial diagnostics, zoonotic disease surveillance, and host‐pathogen interactions. Bioinformatics will be discussed along with new trends in microbial resistance and molecular mechanisms underlying pathogen identification in relation to zoonotic spillover events. By combining artificial intelligence with nanoscale biosensors, microbiology can develop more effective diagnostic platforms, real‐time surveillance tools, and targeted antimicrobials. The standardization of data, the elimination of biosafety concerns, and the development of regulatory frameworks are all essential steps in advancing this cutting‐edge approach to controlling zoonotic disease.

This article is categorized under:

Therapeutic Approaches and Drug Discovery > Nanomedicine for Infectious Disease
Therapeutic Approaches and Drug Discovery > Nanomedicine for Oncologic Disease
Therapeutic Approaches and Drug Discovery > Emerging Technologies

## Graphical abstract

This review highlights how integrating machine learning with nanotechnology enables enhanced diagnostics, personalized treatments, and the prediction of outbreaks for zoonotic diseases, offering a unified framework to advance global health.

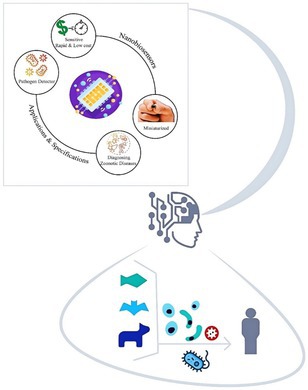

## 1 Introduction

Zoonotic diseases are caused by infectious agents transmitted from animals to humans and account for approximately 60% of all infectious diseases detected worldwide (Esposito et al. 2023; Rahman et al. 2020). A variety of microorganisms have been observed, including fungi, viruses, bacteria, and parasites as causative agents of zoonoses (Sadr, Santucciu, et al. 2025; Ziarati et al. 2022). Animal reservoirs can harbor a variety of pathogens, but humans will not be infected unless several conditions are met, such as optimal environmental conditions (Martin et al. 2018). In the past, zoonotic diseases have led to severe pandemics, such as the Black Death in the 14th century, which resulted in the fatalities of millions of individuals, and the COVID‐19 pandemic most recently (Piret and Boivin 2021). COVID‐19, caused by the SARS‐CoV‐2 virus, has reached over 200 nations, impacting millions of people, incurring billions in costs, and taking nearly 2 million lives since late 2019. Zoonotic disease can quickly strain healthcare systems if not addressed promptly (Aboshosha 2025). Conventional COVID‐19 diagnostic methods face challenges such as the high cost and potential false results of nucleic acid tests like RT‐qPCR, the failure of immunological assays to detect early infections, and the high cost and lack of standardized validation for sequencing methods (Qasem et al. 2021). These have emphasized the persistent and escalating hazard that zoonoses pose to the global economy and public health systems (Sadr, Hajjafari, et al. 2025a).

**FIGURE 1:**
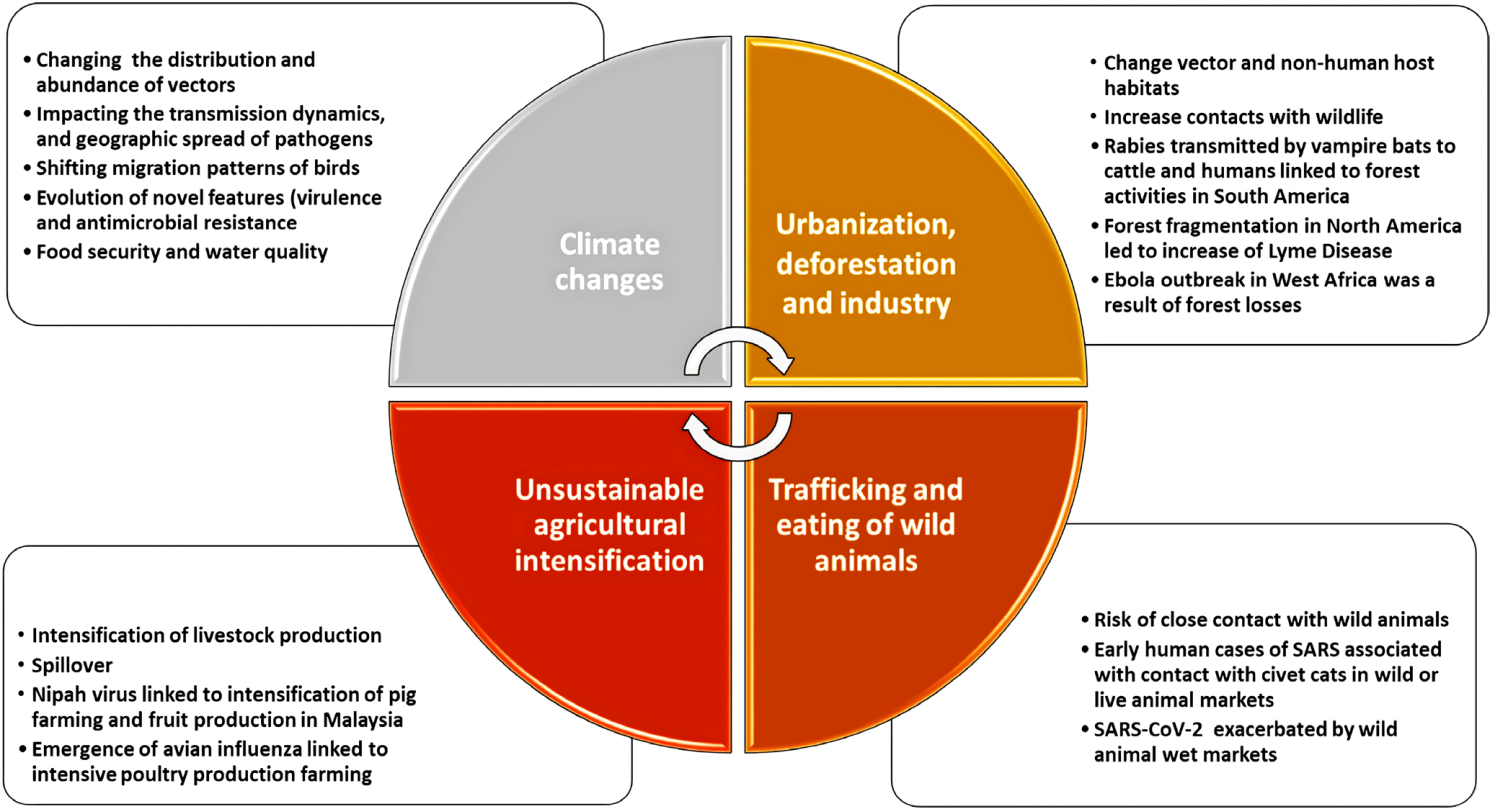
Deforestation, climate change, unsustainable agriculture, and the trafficking of wild animals are some of the factors that contribute to zoonotic outbreaks (Tazerji et al. 2022). This is a CC‐BY reprint.

Several changes in the ecosystem, as well as human behavior, including climate change, habitat invasion, and urbanization, contribute to zoonotic diseases (Figure 1). There is evidence that these diseases can spread through live animal markets and inadequate sanitation in urban environments (Dubey et al. 2023). Trade and travel contribute to the transmission of zoonotic diseases, as evidenced by the 2003 SARS outbreak, which showed that viruses can spread rapidly between countries. A combination of livestock production, industrial farming techniques, and human exposure to zoonotic pathogens makes biosecurity measures an increasing necessity (Bhatia et al. 2024). Generally, losses from zoonotic pandemics affect trade, tourism, and production by $50–100 billion annually. Developing countries face a particular problem when resources diverge from other health priorities. Diseases also cause fear, mistrust, social destabilization, and vulnerabilities in global preparedness systems (Fechner et al. 2023).

The ability to diagnose zoonotic diseases is crucial for effective control of outbreaks, but in resource‐constrained environments, it can be very challenging. Current diagnostic techniques, such as serological assays and polymerase chain reactions (PCR), cannot identify many causal agents and pathogens due to genetic diversity. Considering both time and budget constraints, this process can be very time‐consuming and challenging (Chidzwondo and Mutapi 2024). The lack of advanced infrastructure and specialized knowledge in rural or impoverished areas often leads to uncontrolled disease spread due to ineffective PCR methods. There is a shortage of portable diagnostic devices in rural areas without modern diagnostic facilities (Hobbs et al. 2021). Until reliable and prompt treatment is available at the point of care, zoonotic diseases will continue to spread undetected. Diagnostic tools that can detect multiple diseases must be readily accessible to eliminate this disparity.

The development in machine learning (ML) and nanotechnology has transformed the face of medical diagnostics, thereby overcoming certain limitations posed by conventional techniques. ML is adept at complex data analytics and can recognize patterns hidden from conventional analytics (Sahu et al. 2022). By running ML algorithms on deep epidemiological, clinical, and environmental data, it forecasts epidemics, detects disease patterns, and refines diagnostic methods. The application of supervised learning techniques, such as neural networks and support vector machines, has been shown to be highly efficient for analyzing diagnostic images and detecting abnormalities (Lepakshi 2022). A variety of environmental variables can also be incorporated into ML models to correlate genomic sequences of pathogens and extract reliable conclusions from them. In conjunction with predictive algorithms, it may be possible to pinpoint hotspots for vector‐borne diseases like malaria before the introduction of effective and targeted treatment (Ekundayo 2024).

Nanotechnology, at the atomic and molecular level, enables diagnostics with unprecedented sensitivity and specificity. Nanobiosensors can detect infections at low levels, allowing early diagnosis. Quantum dots, gold nanoparticles, and carbon nanotubes were employed in these sensors to increase their detection capabilities (Barbosa et al. 2021; Markandan et al. 2024). Furthermore, nanotechnology increases drug safety and effectiveness by optimizing drug delivery strategies. Using nanoparticles, drugs can be delivered more efficiently to affected tissues, reducing adverse effects and improving efficacy. It is especially helpful in treating diseases caused by zoonotic organisms, such as tuberculosis, where traditional methods have fallen short (Arshad et al. 2022; Joshi et al. 2021).

By integrating machine learning into nanotechnology, we can significantly improve our ability to diagnose and treat infectious diseases (Khan et al. 2024). The sensitivity and effectiveness of diagnosis are improved by using ML algorithms in conjunction with nanobiosensors, which, coupled with deep learning algorithms, analyze streams of data in real time, enabling automatic pathogen identification, reducing human error, and increasing diagnostic speed (Kokabi et al. 2023). Biomarker identification for certain zoonotic infections by ML‐generated predictive models enables the development of highly effective nanobiosensors (Jindal et al. 2021). Both technologies combine to improve diagnostic accuracy while reducing the cost and complexity of developing diagnostic tools.

These technologies offer tailored diagnostic solutions targeted at individual patients and specific infections. Nanobiosensors integrated with ML algorithms enable rapid on‐site diagnosis, thereby contributing to significant improvements in global health (Dhanalakshmi et al. 2020). This is most paramount in those parts of the globe that have been underserved due to a high degree of zoonotic infections caused by poor health resources. Together, the integration of nanotechnology and ML into diagnostic devices faces challenges in data privacy, the development of laid‐out protocols, and very high development costs. The solution to these challenges is multi‐disciplinary collaboration and investment in research and infrastructure (Khan et al. 2024). The global health community can unleash the full potential of these technologies to address zoonotic diseases by fostering collaborations among governments, academia, and the private sector.

Nanotechnology and ML are increasingly used together to address contemporary technological and scientific challenges, capitalizing on the strengths of both to advance progress in both fields. ML can assist in automating certain steps in nanoparticle identification and creation using trained models, speeding up advancements in the field (Tripathy et al. 2024). A demonstration of ML's influence in nanotechnology is the application of convolutional neural networks to interpret scanning electron microscopy (SEM) images of nanostructures, attaining over 95% accuracy in classifying nanoparticles (Yao and Chen 2023). This degree of efficiency greatly reduced the time and effort required for characterization, enabling the execution of extensive studies at greater speed and precision. These instances highlight ML's revolutionary impact on identifying nanoparticles with specific attributes and streamlining labor‐intensive tasks in nanotechnology (Tulevski et al. 2014).

Integration of ML and nanotechnology greatly enhances disease control through personalized, precise treatments. Machine learning reviews and analyzes large proteomics and genomics datasets to identify key viral targets and refine drug repurposing (Uddin et al. 2024). On the other hand, nanotechnology and 3D bioprinting, specifically, aid in the development of nanoscale drug delivery systems and facilitate in vitro tissue modeling (dos Santos et al. 2021; Rana et al. 2017). Working together, they provide an approach to developing and testing personalized treatments that is rapid, cost‐effective, and individualized (Uddin et al. 2024).

This review evaluates their integration of ML and nanotechnology for diagnosing zoonotic diseases, assessing their current status, and their potential to improve diagnosis in terms of precision, timeliness, and cost. It discusses empirical knowledge regarding zoonotic diseases and their implications for global health. It reviews the gaps in the diagnostic tools, in the knowledge, and in the contribution of zoonotic diseases to health. It reviews the integration of ML and nanotechnology, and then focuses on the specific contribution of each to medical diagnosis. Their integration is evidenced in case studies and examples. The paper concludes with a synthesis of the findings concerning diagnostic tools and technology integration, with the aim of improving global health. The fields of ML and nanotechnology should be easily accessible to health practitioners, health policymakers, and health researchers to address the challenges of zoonotic diseases and facilitate their integration.

## 2 An Overview of Zoonotic Diseases

Among the most critical threats to public health are zoonotic diseases transmitted to people in contact with animals and their products, and anthropozoonotic diseases, also transmissible from human to human (Abebe et al. 2020; Al‐Tayib 2019; Teshome and Addis 2019). Examples of agents that transmit zoonotic diseases include bacteria, viruses, parasites, and fungi (Al‐Sulivany et al. 2024; Rahman et al. 2020). The extent and variety of these pathogenic factors complicate their diagnosis and management and require specialized approaches (Bird and Mazet 2018; Gebreyes et al. 2020; Sharan et al. 2023). Nowadays, as humans keep more pets, the importance of zoonoses in global health systems has become increasingly evident (Suminda et al. 2022). These diseases can easily be transmitted to humans from the environment, pets, livestock, and even wildlife (Hong et al. 2020; Sánchez et al. 2021). As a result, knowledge of the nature and characteristics of these diseases is necessary to formulate effective strategies for diagnosis and treatment (Gwenzi et al. 2022; Steele et al. 2021; Sykes et al. 2022).

Zoonoses are divided into two main categories, direct zoonoses and indirect zoonoses (Ferreira et al. 2021). Concerning direct zoonosis, disease transmission occurs through direct contact with infected animals or their products (Ellwanger and Chies 2021; Noguera Z et al. 2022), such as through bites, scratches, body fluids, or consumption of animal‐derived food, or direct contact with sick animals can transmit diseases (Ellwanger and Chies 2021; Noguera Z et al. 2022). On the other hand, indirect zoonoses are transmitted from animals to humans via vectors or contaminated environments (Recht et al. 2020). These zoonotic diseases are spread through mosquito bites, and examples include malaria and West Nile virus.

### 2.1 Global Importance

The transmission of zoonotic diseases to humans accounts for the majority of new diseases (Galindo‐González 2022; Rohr et al. 2019). It is estimated that up to 75% of new and emerging infectious diseases are being transmitted to humans by animals. More than 60% of human infectious diseases have a zoonotic origin (Rahman et al. 2020). This statistic shows the role of animals in the epidemiology of human diseases (Fenollar and Mediannikov 2018; Weiss and Sankaran 2022; Ye et al. 2020). Climate change, human‐wildlife interactions, human migrations, and land‐use changes all contribute to the spread of zoonoses (Goldstein et al. 2022; Rupasinghe et al. 2022). The spread of certain zoonotic diseases has led to some of history's deadliest pandemics. A clear example is the plague, which killed millions of people during the Middle Ages (Combs et al. 2022). Plague is an infectious disease caused by the bacterium *Yersinia pestis* . Symptoms include fever, weakness, and headache (Barbieri et al. 2020). Usually, this begins 1–7 days after exposure. There are three forms of plague infection that affect different parts of the body and are thus accompanied by different symptoms: plague of the lungs (Lei and Kumar 2022; Nyenke et al. 2023). Infection of the lungs is thus accompanied by difficulty breathing, coughing, and chest pain; their swelling characterizes infection of the lymph nodes; and infection of the blood may be manifested by blackening and death of the tissues (Bourner et al. 2023; Janik et al. 2020; Rosario‐Acevedo et al. 2021). The Coronavirus, COVID‐19, avian influenza, and several other outbreaks of zoonotic diseases are only a few of these. This condition continues to pose a serious threat to global health (Esposito et al. 2023; Horefti 2023; Petrovan et al. 2021; Tabish and Nabil 2022; Tajudeen et al. 2022; Tomori and Oluwayelu 2023).

### 2.2 Challenges in the Diagnosis and Treatment

Due to their complexity, diagnosing and treating zoonotic diseases present many challenges (Gebreyes et al. 2014; Nii‐Trebi 2017). A major challenge lies in the variety of pathogenic agents and their transmission methods (Pal, Tariku, et al. 2024; Pérez‐Lago et al. 2014). Some zoonoses are easily diagnosed, but many others require specialized and complex tests. For example, viruses associated with zoonoses, such as hantavirus or Nipah virus, may have nonspecific early symptoms that are easily mistaken for other diseases (Organization 2014; Rosenberg 2015). This makes a quick and accurate diagnosis difficult. Rabies, for instance, can be transmitted by the bite of an infected animal. In contrast to direct zoonoses, indirect zoonoses are transmitted through vectors or contaminated environments (Cascio et al. 2011; Halliday et al. 2012; Hobbs et al. 2021). To eliminate mosquito vectors of diseases such as malaria and leishmaniasis, comprehensive control measures must be employed (Montenegro Quiñonez et al. 2021; Shaw and Catteruccia 2019; Wilson et al. 2020).

When it comes to zoonoses, there has been a lack of diagnosis and treatment facilities that are needed when diagnosis and treatment are required (Welburn et al. 2015). There is a lack of health infrastructure in rural and remote areas that are exposed to endemic zoonoses for rapid diagnosis and proper treatment. This causes many cases of disease to remain undiagnosed and to spread to wider communities (Hattendorf et al. 2017). Further, treatments for zoonoses are highly diverse with respect to disease type and the pathogenic factors involved (Awaidy and Al Hashami 2020; Joshi et al. 2021). While some zoonotic diseases are treatable with antibacterial or antiviral drugs, others have no effective treatments yet (Głowacka et al. 2018; Karesh et al. 2012; Kilpatrick and Randolph 2012).

Various approaches are being developed and implemented to combat zoonotic diseases (Vrbova et al. 2010). Some of the most important methods in use include advanced warning and monitoring systems (Hassan et al. 2023). These systems predict and identify zoonotic disease incidence by collecting and analyzing biological and epidemiological data. Emerging technologies, like artificial intelligence and ML in data processing, may enhance the speed and precision of diagnosis. Furthermore, it is essential to establish educational programs for at‐risk populations, including farmers, ranchers, and those in contact with wildlife, to mitigate the spread of zoonotic diseases. The advancement of vaccinations and other pharmaceuticals is regarded as a crucial technique for therapy. Effective vaccinations are available for some zoonoses, including rabies and yellow fever, to prevent their transmission within the human population (Carpenter et al. 2022). Much research is needed in immunology and vaccine development to address zoonotic diseases. Therefore, it is important to focus on research and the development of new technologies to reduce these diseases (Ali 2023; Keshavamurthy et al. 2022). A variety of technologies, such as nanotechnology, biosensors, and artificial intelligence algorithms, can identify pathogenic agents early and predict future diseases. Nanotechnology, including nanobiosensors, is one of the new tools that can be used to manage zoonoses effectively in the future (see Table 1) (Kour et al. 2022).

**TABLE 1:** Comparison table of diagnostic techniques of traditional and machine learning + nanotechnology‐based methods based on their speed, cost, scalability, sensitivity, specificity, ease of use, portability, sample volume, adaptability, environmental impact, reliability, data integration, training data, maintenance, and future potential (Khan et al. 2024; Ramalingam et al. 2023; Sadr, Hajjafari, et al. 2025b; Syed et al. 2025; Yin 2025).

Criteria	Traditional diagnostic methods	Machine learning + nanotechnology‐based methods
Speed	Time‐consuming (hours to days)	Rapid (minutes to hours)
Cost	Variable (medium to high)	Potentially lower with scalability
Scalability	Limited	High scalability with automated systems
Specificity	Dependent on the test type	Highly specific due to ML‐driven analysis
Ease of use	Requires skilled personnel	User‐friendly interfaces with automation
Portability	Often laboratory‐bound	Portable devices available with nanotechnology
Sample volume	Large amounts required	Minimal sample size required
Adaptability	Limited to specific diseases	Adaptable to multiple pathogens with re‐training
Environmental Impact	Waste‐producing	Eco‐friendly with biodegradable nanomaterials
Reliability	Subject to human error	Consistent and reproducible results
Data integration	Minimal	Seamless integration with digital health records
Training data	Not applicable	Requires large datasets for machine learning
Maintenance	Frequent calibration needed	Low maintenance due to advanced materials
Future potential	Moderate innovation potential	Continuous improvement through AI development

## 3 Machine Learning

One of the most novel techniques in the medical field includes ML, a subset of Artificial Intelligence (AI), over recent decades (Ahmed et al. 2020; Raschka et al. 2020). With this technology, systems can learn from data and make decisions autonomously without requiring detailed planning (Panch et al. 2018; Xu et al. 2021). Machine learning techniques can be used in medicine to analyze complex datasets and diagnose and predict zoonoses (Barragán‐Montero et al. 2021; Burns et al. 2023; Jordan and Mitchell 2015; Yakimovich 2019). In diseases transmitted through human‐animal interactions, ML is significant for detecting complex patterns and predicting their spread (Talukder et al. 2024; Wardeh et al. 2020). The use of this technology enables faster and more accurate diagnosis and management of infectious diseases (Mathison et al. 2020; Rhoads 2020; Wang et al. 2022).

**FIGURE 2:**
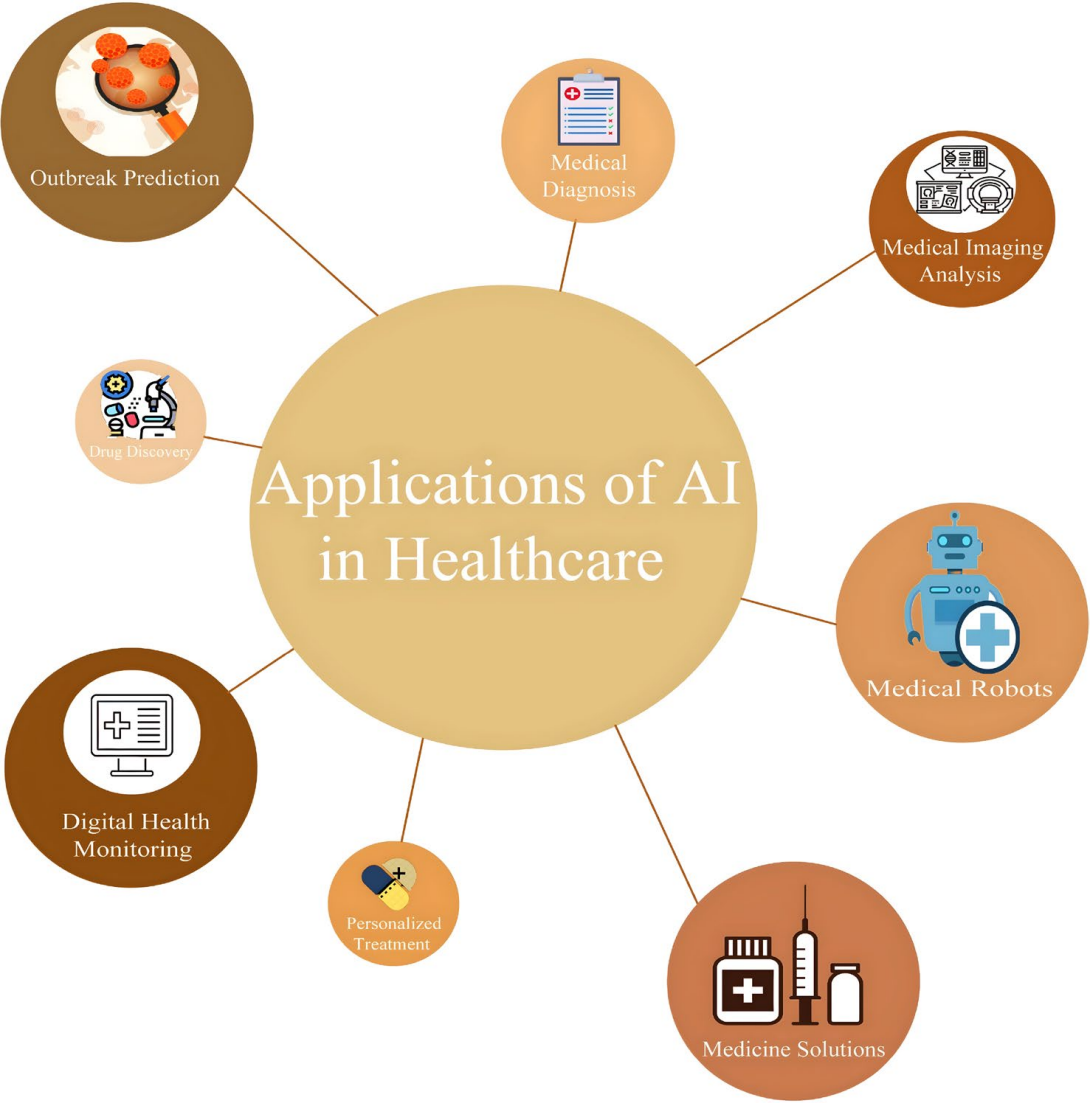
This chart shows the applications of artificial intelligence (AI) in healthcare, including digital health monitoring, drug discovery, new medical solutions, outbreak prediction, medical imaging analysis, medical robots, medical diagnosis, and personalized treatments that can improve an individual's health.

Machine learning is the use of algorithms and statistical models to analyze data and improve system performance through experience. Unlike traditional programming methods that require manual adjustment of each part of the process, ML enables systems to adapt to changes using big data and incremental learning automatically (Guo et al. 2023). This technology uses several types of learning: supervised, unsupervised, and reinforcement. In supervised learning, models learn patterns and relationships from labeled data. In unsupervised learning, the system seeks to identify patterns and clusters in unlabeled data. The reinforcement learning process also uses feedback from the environment to improve the model's performance. Due to their ability to analyze complex and multidimensional data, ML algorithms have become popular tools in medicine for analyzing biological, genetic, and epidemiological data (Ezanno et al. 2021; Peng et al. 2022; Pillai et al. 2022). The demand for intelligent analytics is increasing as large amounts of medical data, including clinical trial results, medical images, and genomics data, become available (Dilsizian and Siegel 2014; Ibrahim and Abdulazeez 2021; Noorbakhsh‐Sabet et al. 2019) (Figure 2).

Diagnosis of diseases is one of the most important applications of ML in medicine (Yoo et al. 2012). Various algorithms are used to analyze medical data, each with its own advantages and limitations (Ngiam and Khor 2019). Among the most popular ML algorithms are Artificial Neural Networks (ANNs), Support Vector Machines (SVMs), and Random Forests (Boateng et al. 2020; Han et al. 2018; Hsieh et al. 2011; Ibrahim and Abdulazeez 2021). In imitation of the human brain, artificial neural networks use multiple layers to process data and can identify complex relationships between inputs and outputs. Cancer and heart disease are two complex diseases for which these algorithms have been highly successful at diagnosis (Kumar, Koul, et al. 2023; Razzak et al. 2020). Deep learning spots subtle patterns in medical images and genetic data, which helps catch cancer early. On the other hand, SVMs and random forests dig through clinical and imaging data to predict heart disease. Using these tools, doctors achieve more accurate results with fewer diagnostic errors (Sharma et al. 2025; Yao et al. 2025).

Machine learning algorithms such as SVMs are also powerful at identifying and classifying diseases (Pisner and Schnyer 2020). With this algorithm, data is divided into categories, and accurate decision boundaries are created in a multidimensional space for medical diagnosis. The accuracy of SVM makes it ideally suited for analyzing medical data, especially for diagnosing diseases with complex, nonlinear characteristics (Abdullah and Abdulazeez 2021; Fatima and Pasha 2017; Senturk 2020). Random forests are among the most widely used algorithms that combine several decision tree models to bring higher accuracy in predicting and diagnosing diseases (Denisko and Hoffman 2018; Dinesh et al. 2024). These algorithms show better performance, especially in cases where the data has noise and disturbance. Random Forests reduce the effect of outliers through ensemble voting, while SVMs optimize class boundaries to minimize misclassification. Studies show both maintain high accuracy and low false‐positive rates even with substantial noise (Dasari et al. 2025).

Predicting the spread of zoonotic diseases is one of the most challenging aspects of zoonotic disease management and control (Kelly et al. 2020; Ogden et al. 2017). To accurately predict the prevalence of zoonotic diseases, detailed, multidimensional analyses are necessary, given their complexity and unpredictable nature (Cardoen et al. 2017; Carlson et al. 2021; Christaki 2015). In this situation, ML can be highly useful. In this technology, epidemiological, environmental, and genetic data are used to identify patterns of zoonotic disease outbreaks (Vilne et al. 2019). For example, supervised learning algorithms can identify new patterns for predicting future outbreaks by analyzing historical data on outbreaks of various diseases and examining environmental variables such as changes in temperature and humidity.

One of the successful applications of ML in predicting the spread of zoonoses is its use in analyzing geographic and environmental data (Mollalo et al. 2018; Peters et al. 2020). These data are complemented by information on vector distribution, weather conditions, and human behavior that influence disease transmission (Kaur et al. 2022; Kondeti et al. 2019). The data can be analyzed using ML, enabling the identification of patterns associated with disease outbreaks. These patterns represent recurring associations among variables connected to disease outbreaks. Examining them enables forecasting high‐risk locations and times, directing focused efforts to reduce transmission and its effects (Santangelo et al. 2023).

**FIGURE 3:**
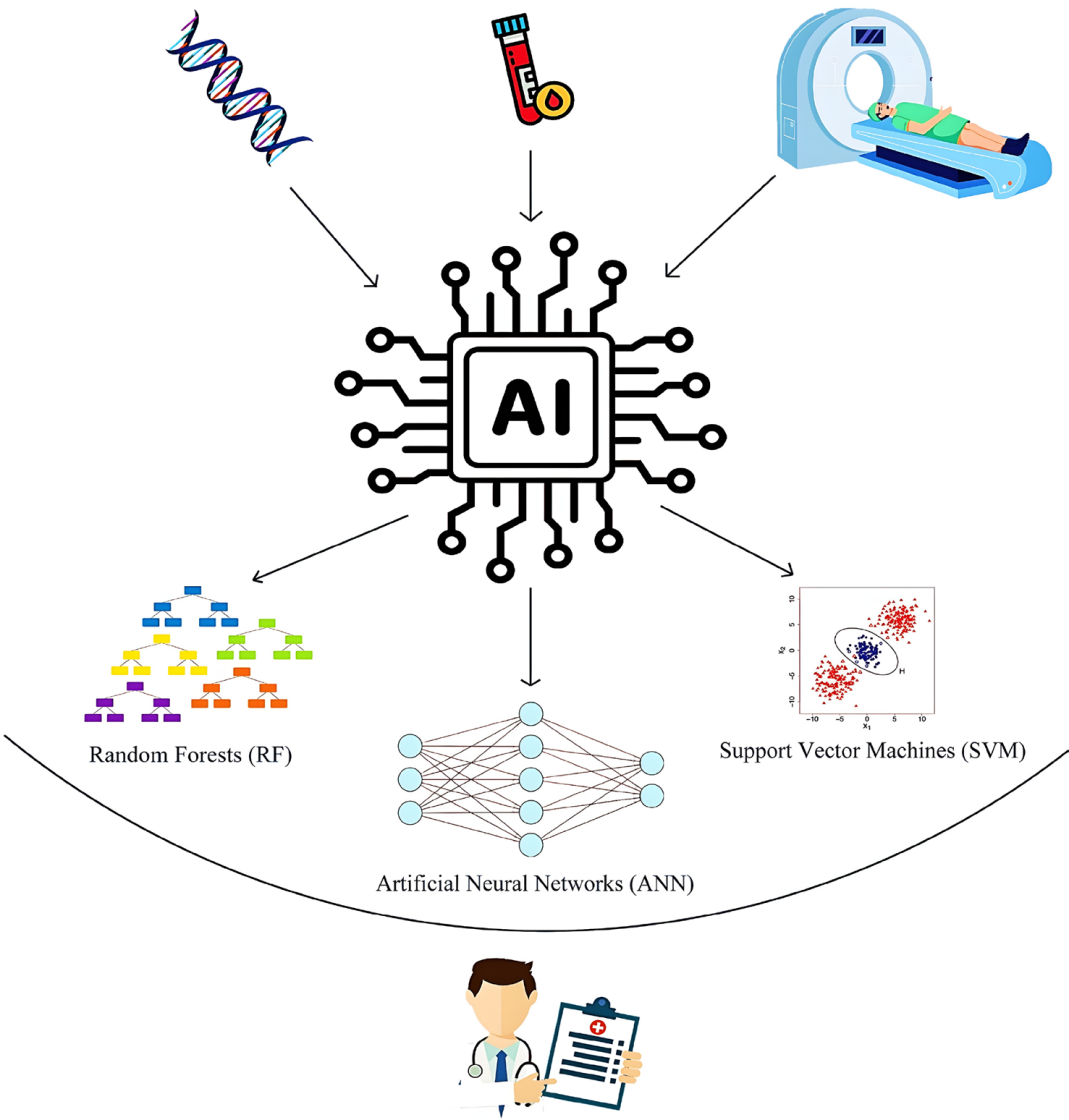
This figure explains how Artificial Intelligence can gather datasets from blood sampling, genetic results, and medical imaging to help clinicians make decisions faster, diagnose more accurately, and predict diseases, offering a glimpse of the future of medical diagnostics. In this regard, artificial neural networks (ANNs), support vector machines (SVMs), and random forests (RFs) can be applied.

This allows early warnings to be issued for at‐risk areas (Hao et al. 2022; Ito et al. 2024). The use of this approach has also helped prevent the spread of various other diseases, including West Nile virus (see Figure 3 and Table 2).

**TABLE 2:** This table overviews popular machine learning algorithms applied to zoonotic disease diagnostics, highlighting their unique strengths, potential drawbacks, and real‐world applications.

Algorithm	Strengths	Limitations	Examples of applications in diagnostics
Support Vector Machines (SVM)	High accuracy for binary classification, effective in high‐dimensional spaces, and resistant to overfitting.	It requires careful parameter tuning, is not well‐suited to large datasets, and is sensitive to noise.	Classification of zoonotic pathogens based on genomic or proteomic data; detecting TB or brucellosis using medical images.
Random Forest (RF)	Handles large datasets well, robust to overfitting, and interpretable through feature importance.	Computationally intensive for large‐scale problems, less effective with sparse datasets.	Identifying zoonotic disease outbreaks based on environmental and demographic data; classifying infectious agents in samples.
Neural Networks (NN)	Capable of modeling complex patterns and relationships, highly flexible, and supports end‐to‐end learning.	It requires large datasets and significant computational resources; with small datasets, there is a risk of overfitting.	Disease risk prediction using patient health records; detecting zoonotic viruses like Nipah or H5N1 from imaging or genomic data.
Convolutional Neural Networks (CNN)	Exceptional at processing image data, automatically extracts features, and is suitable for spatial pattern recognition.	It requires large labeled datasets but is computationally expensive and less interpretable.	Diagnosing zoonotic diseases through radiological imaging (e.g., chest x‐rays for zoonotic TB); analyzing histopathological slides for parasite detection.
Recurrent Neural Networks (RNN)	Excellent for sequential or time‐series data, captures temporal dependencies, and supports dynamic analysis.	Difficulty in training due to vanishing gradient problems; not ideal for very long sequences.	Tracking zoonotic disease outbreaks using time‐series epidemiological data; analyzing gene expression patterns in zoonotic infections.
Gradient Boosting Machines (e.g., XGBoost, LightGBM)	High accuracy, handles imbalanced datasets, and is robust to missing data.	Computationally intensive; can overfit without proper regularization.	Predicting zoonotic disease susceptibility using clinical and environmental factors; identifying critical biomarkers.
K‐Nearest Neighbors (KNN)	Intuitive and straightforward; effective for small datasets and non‐linear relationships.	It is sensitive to noise, computationally expensive for large datasets, and requires careful choice of K.	Classifying zoonotic pathogens in small datasets; predicting disease status based on symptoms or lab test results.
Bayesian Networks (BN)	The probabilistic approach handles uncertainty well, is interpretable, and incorporates prior knowledge.	Computationally expensive for large networks; less effective with incomplete data or poorly chosen priors.	Assessing zoonotic disease risks in populations using probabilistic modeling, combining lab test results with prior knowledge.
Decision Trees (DT)	Easy to interpret and implement; performs well on small to medium‐sized datasets.	Prone to overfitting and instability; less effective with noisy or complex data.	Initial screening for zoonotic diseases in veterinary settings; identifying likely sources of zoonotic infection.
K‐Means Clustering	Simple and effective for unsupervised clustering; works well for data grouping.	Requires pre‐specification of the number of clusters (K); sensitive to initialization and outliers.	Grouping zoonotic pathogens based on genetic similarity; clustering environmental samples to detect disease reservoirs.
Principal Component Analysis (PCA)	Reduces dimensionality, aids visualization, and highlights key patterns in data.	Limited interpretability and can lose information during dimensionality reduction.	Identifying dominant genetic or environmental factors associated with zoonotic diseases.
Autoencoders	Useful for anomaly detection, feature extraction, and reducing noise in data.	Requires large datasets for training and can suffer from reconstruction errors.	Detecting anomalies in zoonotic disease diagnostic images or genomic data.

### 3.1 VETSCAN IMAGYST

Regular fecal analysis is crucial for monitoring parasitic infections in domestic animals (Colombo et al. 2022; Giannelli et al. 2024). The accuracy of these analyses, defined by their sensitivity and specificity, depends significantly on the sample preparation techniques and the level of training and experience of the personnel examining the slides (Inácio et al. 2021; Nourollahi Fard et al. 2024). The VETSCAN IMAGYST system is a comprehensive diagnostic tool that combines a sample preparation device with a commercially available scanner and sophisticated analytical software. The VETSCAN IMAGYST system allows for automatic identification, classification, and diagnosis of parasite eggs on microscopic fecal slides using a cloud‐based deep learning algorithm (Cringoli et al. 2021; Parija and Poddar 2023). This system provides a simple technique for detecting parasitic disease in fecal samples, requiring minimal examiner experience (Owens et al. 2023). The VETSCAN IMAGYST system employs a deep learning‐based image analysis model trained on expert‐classified fecal samples. During training, experts prepare fecal slides using the VETSCAN IMAGYST centrifugal flotation method, scan them with an automated microscope, and then annotate the images to build a top‐notch labeled dataset (Nagamori et al. 2021b). The algorithm sorts through these images and spots parasite eggs with impressive accuracy, with a sensitivity ranging from 75.8% to 100% and a specificity ranging from 93.1% to 100%. These results align closely with those of human experts, strongly demonstrating the system's reliability for in‐clinic diagnostics (Nagamori et al. 2024; Nagamori et al. 2021a; Steuer et al. 2024).

It has been shown to reliably detect four parasites: *Ancylostoma*, *Toxocara*, *Trichuris*, and members of the Taeniidae family in stool samples (Nagamori et al. 2020, 2021b). The VETSCAN IMAGYST system incorporates three key elements: a sample preparation device, an automated microscope scanner, and a data processing algorithm using deep neural networks. Its convolutional neural network uses deep learning to detect objects by extracting the most discriminative features between classes (Oliver Broome 2024).

Each detected object is assigned a likelihood score by the system, which corresponds to a specific parasite genus or group that the algorithm has been trained to recognize. This sets up a sophisticated model trained on expert‐classified samples that differentiates between parasitic eggs and non‐parasitic artifacts on fecal flotation slides. After adequate training, the model is tested on diverse datasets to ensure it generalizes well to similar contexts. In a single study, VETSCAN IMAGYST showed a diagnostic sensitivity of 75.8% and a specificity of 97.0% compared with expert review for *Giardia* cysts. There is also a common weakness that most object detection algorithms share, which this system also exhibits: correctly positioning small objects like *Giardi*a cysts and correctly differentiating between them (Nagamori et al. 2021b). In contrast, performance continues to improve with additional training. In recent findings, eliminating samples with ≤ 10 cysts per gram (CPG) increased the sensitivity to 95.2%. Traditional microscopy is especially challenging for detecting such low concentrations of cysts. In the same study mentioned above, the system efficiently detected both collapsed and intact *Giardia* cysts.

It is highly recommended that symptomatic pets and animals newly brought into households undergo *Giardia* testing, as many infections are asymptomatic. Analysis of fecal samples has shown that the VETSCAN IMAGYST system is highly accurate for parasite detection. No matter how experienced an examiner is, centrifugal flotation can also provide results in as little as 10 min. It is a suitable alternative to the conventional passive flotation method in veterinary diagnostics due to its efficacy. As well as providing deep‐learning algorithms, the system offers several other benefits (Kanski et al. 2024; Nagamori et al. 2024). Currently, the VETSCAN IMAGYST is the only automated diagnostic tool in veterinary medicine using this sophisticated technology. Furthermore, images and reports are stored in the cloud for collaboration and knowledge sharing among veterinarians, parasitologists, and researchers (Capuozzo et al. 2024). As experience continues, the system should become more effective and provide a useful tool for veterinarians to perform fecal examinations (Ul Haq et al. 2024).

**FIGURE 4:**
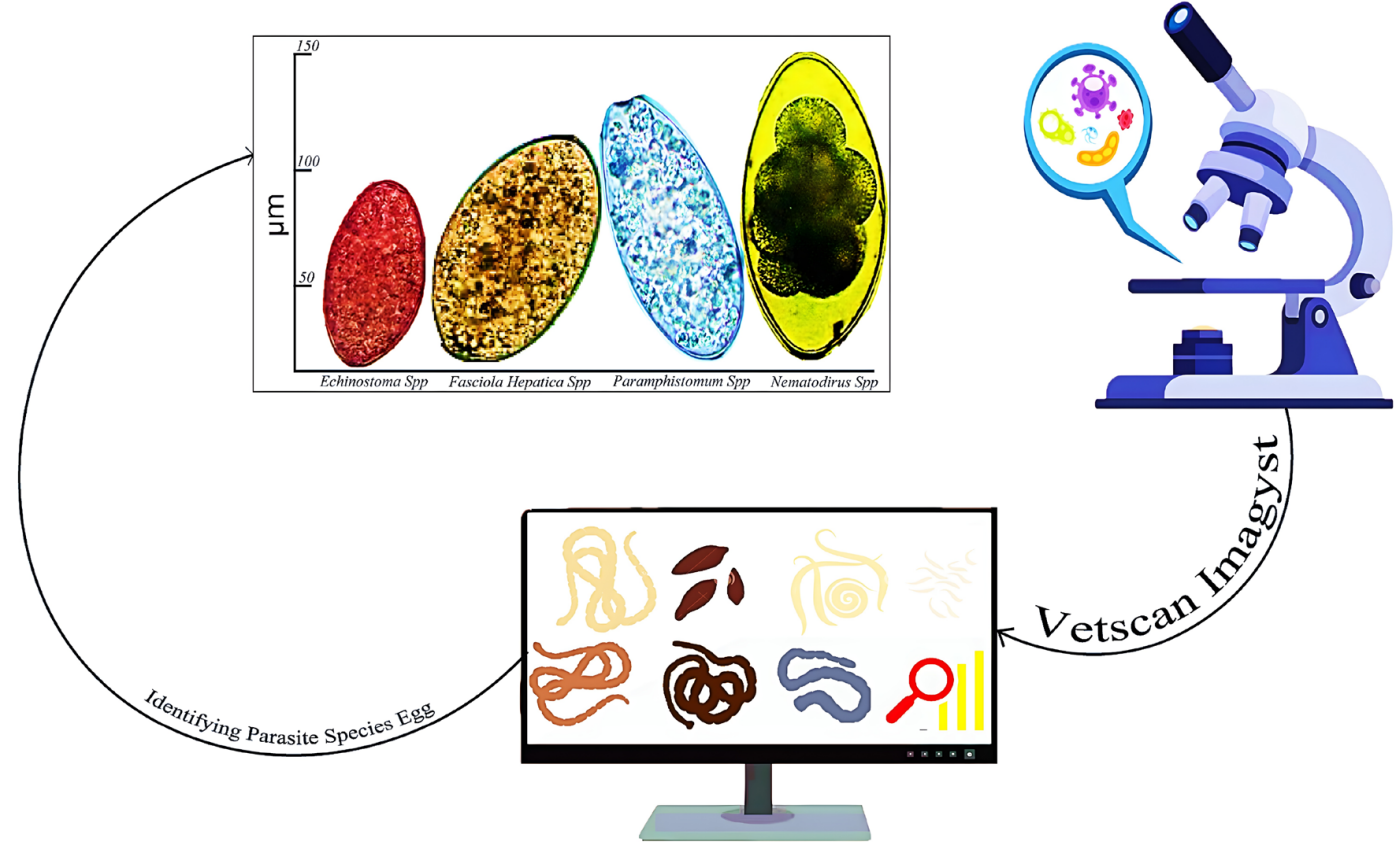
This picture shows “Vetscan Imagyst,” which offers multiple applications for companion diagnostics in a single platform, including differential diagnoses between parasite’ eggs and digital cytology image transfer.

Even now, there are still some limitations with the VETSCAN IMAGYST system. For instance, only a limited area of a sample can be scanned, precluding analysis of the outer edges of a coverslip, where parasite eggs are often located. This is particularly apparent when too much solution is on the slide and the eggs in the periphery of the solution are not found. Like other object‐detection systems, the small objects cannot be accurately differentiated. The software can be further improved to handle such problems, and newer versions can be developed with these modifications (Figure 4). Future work might involve checking the system's performance against other image analysis platforms to confirm and improve its diagnostic reliability. Comparative analyses would help identify the strengths of different systems. This could improve detection efficiency and expand diagnostic applications in veterinary medicine.

## 4 Nanotechnology

There are numerous applications of nanotechnology across several disciplines, especially in biological, veterinary and medical sciences, making it one of the most advanced and innovative fields of science today (Haleem et al. 2023; Sahu et al. 2021). It can bring tremendous changes in disease diagnosis, treatment, and management (Hajjafari, Sadr, Santucciu, et al. 2024; Sadr, Poorjafari Jafroodi, et al. 2023). Due to the minute size of nanomaterials, nanotechnology can address challenges in medicine, drug delivery, and biotechnology and provide innovative solutions (Adir et al. 2020; Sindhwani and Chan 2021). Nanotechnology refers to the science and technology of nanometer‐scale dimensions, ranging from about 1 to 100 nm (El‐Sayed and Kamel 2020; Pramanik et al. 2020). Materials at the nanoscale exhibit entirely distinct features from those at the macroscale due to differences in their physical, chemical, and biological characteristics (Khoobchandani et al. 2020; Sadr, Lotfalizadeh, et al. 2023). As a result, nanotechnology is now being used in a wide range of industries, including electronics, the environment, biological sciences, and medicine (Sim and Wong 2021). Nanotechnology is providing new ways in medicine for diagnosis and treatment with higher accuracy through techniques such as nanoparticles, nanobiosensors, and complex nanostructures (Su et al. 2020).

A nanotechnology approach can be used to manipulate and engineer materials through control and engineering at the atomic and molecular levels (Mazayen et al. 2022; Sarella et al. 2024). Biological systems can be precisely controlled, allowing engineers and bioscientists to construct more complex systems that can interact directly with living things (Khan, Sadia, et al. 2022). In particular, nanoparticles and nanostructures have been used for targeted drug delivery to specific sites in the body, for the identification of diseased cells, and even for tissue repair (Jin et al. 2020; Kirtane et al. 2021). This is one of the most promising technologies today due to nanotechnology's multiple capabilities.

### 4.1 Nanobiosensors

One example of the vast potential of nanotechnology in biology is the development of nanoparticles and nanobiosensors for disease diagnostics (Deng et al. 2020; Ukhurebor et al. 2022). Nanoparticles may interact with and bind biomolecules such as proteins, DNA, and RNA due to their small size and large surface area (Abarca‐Cabrera et al. 2021; Hajjafari, Sadr, Rahdar, et al. 2024; Ma et al. 2021). This feature enables nanoparticles to serve as carriers of identification molecules and play a vital role in early disease diagnosis. For example, metal nanoparticles, such as gold and silver, are widely used in the construction of nanobiosensors for accurate and rapid diagnosis of infectious diseases and cancers (Chinchulkar et al. 2023).

By combining nanomaterials and biomolecules, nanobiosensors can detect various biological compounds (Jain et al. 2021). The molecular sensors can detect subtle changes in molecular structure and provide detailed information on overall health (Misra et al. 2022). Nanobiosensors exhibit high sensitivity and can detect even very small changes in a body's biochemical composition (Shand et al. 2022). In the early stages of cancer, nanobiosensors can detect biomarkers that correspond to the most effective treatment plans (Khazaei et al. 2023). Nanobiosensors' capabilities make them powerful diagnostic tools for disease detection.

### 4.2 Targeted Drug Delivery

Science has increasingly used nanotechnology to deliver drugs targeted to specific tissue types using nanotechnology (Shah et al. 2021; Zhang et al. 2018). The term “targeted drug delivery” refers to delivering a drug directly to the site of disease within the body without harming healthy cells (Hu et al. 2018). Using this method to enhance therapies may decrease side effects and boost efficacy (Saeedi et al. 2019). Nanoparticles are a valuable tool in this sector due to their tiny size and ability to transport medicines (Tewabe et al. 2021). Certain nanoparticles may act as pharmaceutical carriers, delivering medications directly to malignant tumors or other diseased tissues (Dos Santos Ramos et al. 2018; Öztürk et al. 2018). Specially designed nanoparticles can deliver drugs to the right location at the right time to achieve targeted drug delivery (Yetisgin et al. 2020). Cancer and other chronic diseases can benefit greatly from this method. There are many types of nanoparticles that can deliver chemotherapy drugs, such as lipids and polymers (Zahin et al. 2020). These nanoparticles deliver drugs directly to cancer cells while preventing healthy cells from exposure by recognizing cancer cells and binding to them.

**FIGURE 5:**
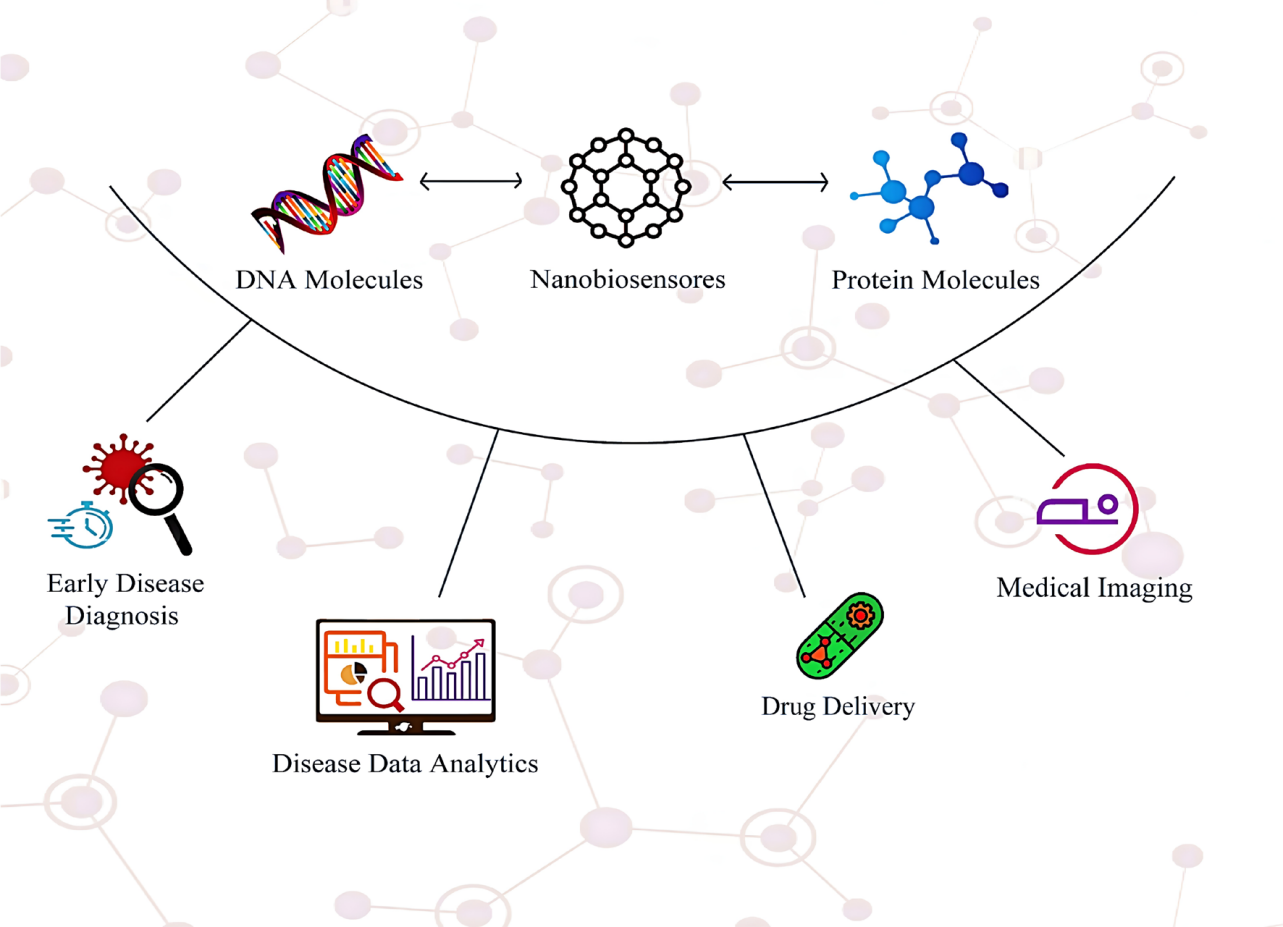
This illustration concept demonstrates the critical role of nanotechnology in the application of DNA and protein molecules by emphasizing the benefits of advanced nanomaterials in drug delivery, medical imaging, early disease diagnosis, and disease data analytics.

Using nanotechnology, scientists can design smart nanoparticles that respond automatically to environmental changes and disease conditions (Maghsoudnia et al. 2020). Drugs can be released at the right time when changes in temperature, pH, or specific concentrations of molecules activate smart nanoparticles. pH‐sensitive nanoparticles, for example, are activated by acidic environments such as tumors, releasing their drugs only when cancer cells are present (Sun et al. 2023). These features can enhance the treatment's effectiveness and reduce side effects resulting from the drug's systemic release (Gottardo et al. 2021). In addition, smart nanoparticles can be used as tools for simultaneous imaging and disease diagnosis (Sanzari et al. 2019). These so‐called “tropic” nanoparticles can deliver the required medicine to the affected area while detecting the exact location of the disease. This technology has raised great hopes, especially for treating cancer and neurological diseases. By combining diagnosis and treatment into a single system, smart nanoparticles can improve treatment outcomes and reduce diagnostic and treatment time (Figure 5) (van der Meel et al. 2019).

## 5 Nanobiosensors Applications

### 5.1 Bacterial Infections

Bacterial zoonotic diseases can be transmitted to humans through direct contact with infected animals, consumption of infected animal products, or insect vectors (Cantas and Suer 2014; Helmy et al. 2017; Laroche et al. 2018; Sanyaolu et al. 2016). Many of these diseases are highly dangerous and pose major challenges to public health, especially in rural areas. One of the most important bacterial zoonotic diseases is plague, caused by *Yersinia pestis* (Barbieri et al. 2020; Yang et al. 2023). This bacterium is normally transmitted to humans via fleas that are carrying the disease (He et al. 2021; Lei and Kumar 2022). Plague caused deadly epidemics worldwide in the distant past and remains endemic in some regions (Høiby 2021; Mas Fiol et al. 2023). The three forms of plague are bubonic, pneumonic, and septicemic; each enters the human body via a different route and causes different complications (Ansari et al. 2020; Evans 2022; Glatter and Finkelman 2021; Nelson et al. 2020; Rabaan et al. 2019; Rosario‐Acevedo et al. 2021; Vallès et al. 2020; Venugopal and Pechous 2024). The rapid transmission of this disease and the high risk of widespread outbreaks make plague one of history's most important bacterial zoonoses.

Another important zoonotic disease is brucellosis, caused by the *Brucella* spp. (Akhtardanesh et al. 2025; El‐Sayed and Awad 2018). This bacterium enters the human body through unpasteurized milk and dairy products, contact with infected animal tissues, or inhalation of bacterial aerosols (Khairullah, Kurniawan, Puspitasari, et al. 2024; Zhou et al. 2020). Animals such as cows, sheep, goats, and pigs can carry this disease, and its symptoms primarily affect the reproductive system. While brucellosis in humans has a protracted course of disease characterized by symptoms such as fever, night sweats, fatigue, and muscle and joint pain, it may persist chronically (Dadar et al. 2021; Hull and Schumaker 2018; Moriyón et al. 2023). Appropriate health measures in animal husbandry and prevention in the human population can control this disease.

Anthrax is also a zoonotic bacterial disease that is caused by *Bacillus anthracis* (Alam et al. 2022). Normally, it is transmitted to humans through contact with infected animals or by inhaling spores of the bacteria (Doganay et al. 2023; Sardar et al. 2023). The three forms of this disease are cutaneous, gastrointestinal, and pulmonary, each with severe implications (Khairullah, Kurniawan, Effendi, et al. 2024; Ogunleye et al. 2024). The pulmonary type is especially deadly, and the spores can survive for a long time in the environment (Savransky et al. 2020). These characteristics make anthrax a serious threat in terms of biosecurity, too, because it has the potential to be used as a biological weapon.

Leptospirosis caused by *Leptospira* spp. is another bacterial zoonosis, especially common in tropical and subtropical climates, and is transmitted to humans through contact with water contaminated with animal urine, such as rats and other rodents (López‐Robles et al. 2021; Md‐Lasim et al. 2021; Nova et al. 2020; Sarabandi et al. 2025). Leptospirosis can cause symptoms such as fever, severe headache, muscle pain, jaundice, and, in severe cases, kidney, liver, or internal bleeding. It can occur as an epidemic in areas with poor water and sewage sanitation, and its control requires extensive health measures (Cilia et al. 2021; Hernández‐Rodríguez and Trujillo‐Rojas 2022; Setyaningsih et al. 2022).

Another important bacterial zoonosis is campylobacteriosis caused by *Campylobacter* spp., especially *Campylobacter jejuni* , and is mostly transmitted by contaminated chicken meat or unpasteurized water and food (Amin et al. 2023; Nakhaee and Hafez 2023; Zhang and Sahin 2020). Campylobacteriosis is usually associated with diarrhea, fever, and abdominal pain, and in some cases can lead to serious complications, such as Guillain‐Barré syndrome (Al‐Naenaeey et al. 2022; Ammar et al. 2021). Campylobacteriosis is one of the most important causes of bacterial gastroenteritis worldwide, and proper management of slaughter processes and food preparation plays a vital role in its prevention (Asmat and Khan 2020; Olvera‐Ramírez et al. 2023).

One of the most critical applications of nanobiosensors in diagnosing bacterial zoonotic diseases is the use of metal nanoparticles, such as gold and silver nanoparticles (Ahangari et al. 2023; Bansal et al. 2016; Rios et al. 2024). Due to their outstanding optical and electrochemical properties, these nanoparticles are used to develop high‐sensitivity nanobiosensors for rapid detection of bacteria such as *Brucella* and *Leptospira* (Castillo‐Henríquez et al. 2020; Dawood et al. 2023; Ghafouri et al. 2023). Nanobiosensors based on Au nanoparticles have been used for the diagnosis of brucellosis due to their ability to amplify the electrochemical signal (Ahangari et al. 2022; Hashemzadeh et al. 2023; Rahi et al. 2015; Sotnikov et al. 2020; Vakili et al. 2021). Such nanobiosensors selectively detect antigens produced by bacteria in the infected body of an animal or a human. Besides metal nanoparticles, nanobiosensors based on carbon nanotubes have also been considered for the diagnosis of zoonotic bacterial diseases (Saklani et al. 2024; Sharma et al. 2020). Carbon nanotubes enable accurate pathogen detection due to their high surface area and strong adsorption of target molecules. For example, nanobiosensors based on carbon nanotubes can detect leptospirosis with very high accuracy (Bhardwaj et al. 2017). This technology helps identify the surface proteins of disease‐causing bacteria, thereby preventing the spread of disease in humans and animals.

Besides carbon nanotubes, polymer nanofibers are other nanomaterials that have been used in some research to diagnose zoonotic bacterial diseases using nanobiosensors (Ghasemi et al. 2022; Jonidi Shariatzadeh et al. 2024; Yang et al. 2024). Special structures of this kind of nanofiber have led to their high ability to absorb biological molecules (Horne et al. 2020; Pebdeni et al. 2022). Specifically, diagnosing bovine tuberculosis uses this nanofiber‐based biosensor, which can detect it at early stages via adsorption of *Mycobacterium bovis* proteins. Another type of nanobiosensors employed in the diagnosis of bacterial zoonotic diseases is quantum dot‐based nanobiosensors (Shanehsaz et al. 2013; Stanisavljevic et al. 2015). Quantum dots have been used in the detection of biomolecules at very low concentrations due to their unique optical properties (Nikolaev et al. 2024; Tang et al. 2008). These nanobiosensors are employed in the diagnosis of diseases such as brucellosis. For instance, cadmium sulfide‐based quantum dots, due to their high fluorescence, can easily detect Brucella bacteria in complex biological samples such as blood or urine. Graphene‐based nanobiosensors are among the technologies widely used for the diagnosis of zoonotic bacterial diseases. Because of its unique electrical and mechanical properties, graphene enables rapid and precise pathogen detection (Sharma et al. 2020). For instance, graphene nanobiosensors are effective for diagnosing leptospirosis because they can detect small changes in the concentration of biological molecules produced by *Leptospira* spp. (Taheri et al. 2024). Moreover, nanobiosensors based on magnetic nanoparticles also play an important role in the diagnosis of bacterial zoonotic diseases. Because of their magnetic properties, magnetic nanoparticles can be easily controlled and absorbed by an external magnetic field. This feature enables nanobiosensors to absorb and detect target molecules, such as bacterial surface proteins, with excellent precision. For example, the detection of bovine tuberculosis rapidly using magnetic nanobiosensors, which technology reduced the detection process from weeks to a few hours (Pashchenko et al. 2018).

Conducting polymer‐based nanobiosensors is another vital category for diagnosing zoonotic bacterial diseases (Prajapati and Kandasubramanian 2019). Due to their unique electrical properties, these polymers can transmit electrical signals to diagnostic systems, enabling rapid detection of bacterial pathogens (Kuznetsova et al. 2023). Conductive polymers can also bind to biomolecules, such as antibodies, thereby increasing the sensitivity of nanobiosensors designed to detect diseases. DNA‐based nanobiosensors are among the most important modern technologies for detecting zoonotic bacterial diseases. Using DNA or RNA strands that bind to bacterial target molecules, these sensors can quickly and accurately identify pathogenic genes (Tessaro et al. 2022). For example, DNA‐based nanobiosensors detect antibiotic resistance genes in zoonotic bacteria such as Brucella and *Leptospira*, which can help manage treatment and prevent disease outbreaks.

### 5.2 Viral Infections

Viral zoonosis diseases represent some of the major health hazards. Since viruses can be transmitted from animals to humans, these diseases pose significant challenges for control and prevention due to the direct or indirect ways the disease agent may be transmitted from animal sources (El Amri et al. 2020). These diseases vary widely in the types of viruses involved, modes of transmission, and death rates. It includes diseases such as bird flu, Crimean‐Congo fever, Nipah virus, rabies virus, and viruses of the coronavirus family (Al‐Tayib 2019; Tomori and Oluwayelu 2023; Venkatesan et al. 2010). These diseases have always interested researchers and international health organizations due to their high potential to cause pandemics and epidemics. Avian influenza is one of the most prominent zoonotic viral diseases that has caused widespread concern in recent years (Kim et al. 2016; Li et al. 2019; Naguib et al. 2019; Widdowson et al. 2017). Viruses belonging to the family Orthomyxoviridae cause this infection; subtypes include H5N1 and H7N9 (Nuñez and Ross 2019; Su et al. 2017; Wang et al. 2020). Avian influenza viruses are usually transmitted to humans through direct contact with an infected bird or by consumption of its diseased products. This malady has become a very serious global threat not only because of its high birds‐to‐human mortality rate but also owing to its pandemic creation mutation potential (Rafique et al. 2023; Tang et al. 2024). For instance, the H5N1 subspecies, identified in Hong Kong in 1997, has killed many birds worldwide, and deaths among humans have also been reported (Adesola et al. 2024; Bi et al. 2015; Gauthier‐Clerc et al. 2007). This virus is more frequently transmitted from birds to humans, most especially in areas where people are in close contact with wild birds or livestock. One of the major challenges in managing the disease is that these viruses have a high genetic mutation rate and are resistant to existing treatments (Li et al. 2010; Petersen et al. 2024).

Crimean‐Congo fever virus is another important zoonotic viral disease that poses a serious threat to human and animal health (Kalal 2019; Shahhosseini et al. 2021). It is a member of the Bunyavirus family and is transmitted to humans via ticks. It may also be transmitted from human to human through direct contact with blood or body fluids of an infected person (Garg et al. 2024; Wahid et al. 2019). Given its high mortality rate, ranging from 10% to 40% in severe cases, and its rapid spread, this virus is one of the health priorities in many regions of the world, especially in Africa, Asia, and Europe (Ahmed et al. 2022; Aslam et al. 2016; Bernard et al. 2022; Dreshaj et al. 2016). Among the problems associated with this virus is that early diagnosis is difficult because it presents general symptoms at the onset, as with many viral diseases, including fever and headache (Abdiyeva et al. 2019; Health and Welfare 2010). This virus is especially prevalent in rural areas and areas where livestock are widely kept. Ticks are the main vectors of this virus, while cattle, sheep, and goats serve as hosts for infected ticks, and humans contract the virus through contact with such ticks or animal products that harbor it (Chinikar et al. 2009; Mahzounieh et al. 2012).

Nipah virus is a zoonotic virus that has attracted global attention due to rare but fatal outbreaks in regions of Asia, such as Malaysia and Bangladesh (Amaya and Broder 2020; Bruno et al. 2022). It belongs to the Paramyxoviridae family and is probably transmitted to humans by fruit bats (Faus‐Cotino et al. 2024; Pelissier et al. 2019). In Malaysia, it was first reported in 1998, and numerous outbreaks have occurred since then (Chua 2003; Looi and Chua 2007; Soman Pillai et al. 2020). Besides direct contact with infected animals, Nipah virus can also be spread by people infected with it (Mathew et al. 2021). In advanced stages of the disease, severe headaches, fever, and encephalitis can occur, and in the worst cases, death can result (Tennakoon and Wijesundera 2023). Considering that no treatment has been established for this disease as of yet, the most practical precautions to avoid infection are good hygiene practices and limiting contact with infected animals (Broder et al. 2013; Hassan et al. 2024).

Another globally important zoonotic viral disease is rabies (Manjunatha et al. 2023; Rupprecht et al. 2022). Rabies virus belongs to the family Rhabdoviridae, which is usually transmitted to humans by bites from infected animals, especially dogs (Gholami and Alamdary 2020; Leon et al. 2021). This virus is highly virulent, and if symptoms develop, it almost invariably causes the infected individual's death. Rabies is a zoonotic disease that is generally more common in rural areas and countries where access to broad vaccination and stray animal control is limited (Acharya et al. 2022; Amoako et al. 2021; Samad et al. 2024). Although the rabies vaccine is very effective for prevention, thousands of people still die every year due to a lack of access to the vaccine or a lack of timely knowledge (Khairullah et al. 2023; Makovska et al. 2021).

The spread of diseases such as SARS, MERS, and COVID‐19 has also heightened the prominence of Coronaviridae viruses as zoonotic agents (Al‐Salihi and Khalaf 2021; Chakraborty et al. 2020; Contini et al. 2020). There is a great likelihood that these viruses can be transmitted through contact with wild animals, such as bats (Chathappady House et al. 2021; Dhama et al. 2020; Peeri et al. 2020). As an example, in 2002, SARS was likely spread by some species of cats and bats (Brüssow 2023). Additionally, Saudi Arabia struggled with MERS in 2012, which is spread between humans and camels (Azhar et al. 2014; Khalafalla et al. 2015; Sazmand and Nourian 2023). These viruses rapidly became global scourges due to their high ability to transmit from person to person and cause severe respiratory diseases (Bardhan et al. 2023; Horefti 2023; Qiu et al. 2023).

Yellow fever is another zoonotic viral disease that is spread worldwide (Aliaga‐Samanez et al. 2022). Flaviviridae, a family of viruses, cause yellow fever, which is transmitted to humans by infected mosquitoes (Silva et al. 2020). A high number of people are infected with yellow fever every year in tropical Africa and South America (de Oliveira Figueiredo et al. 2020; Hale 2023; Oyono et al. 2022; Pal, Geleto, et al. 2024). As such, there is an effective vaccine for the disease, but large populations of people in poverty‐stricken areas do not have access to the vaccine; therefore, this disease causes tremendous deaths in these regions due to severe complications (Gianchecchi et al. 2022; Sacchetto et al. 2020; Tuells et al. 2022).

The other zoonotic viruses transmitted by mosquitoes are Zika and Chikungunya, which have become widespread in tropical and subtropical regions (Côrtes et al. 2023; Kazmi et al. 2020; Weaver et al. 2020). The Zika virus gained huge importance in 2015, being responsible for severe effects among pregnant women and causing defects to the fetus (Martins et al. 2021; Rasmussen & Jamieson, 2020; Teixeira et al. 2020). Like many zoonotic viruses, these viruses require extensive preventive measures, such as mosquito control and vaccination, due to their high potential to cause megapandemics (Delrieu et al. 2023). Given the wide range of zoonotic viral diseases and the lack of access to rapid, accurate diagnostic systems, controlling and preventing these diseases remains a major challenge (Adam and Jassoy 2021; Nikookar et al. 2020; Socha et al. 2022). Therefore, for early detection, developing advanced technologies such as nanobiosensors enables quick, accurate diagnosis. Advanced nanomaterials, along with biological systems, could enhance the detection of viruses at very low concentrations, and, as a result, nanobiosensors could be an important tool for the diagnosis and control of zoonotic viral diseases.

Several advantages can be gained from nanobiosensors in diagnosing zoonotic viral diseases. Nanobiosensors enable detection of viruses at the earliest stages of infection (Kaya et al. 2020; Ramakrishnan et al. 2021). Accordingly, gold nanomaterials can be used for synthesizing nanobiosensors with the potential to rapidly distinguish viruses of the Coronaviridae family, namely SARS, MERS, and COVID‐19 (Alhalaili et al. 2020; Antiochia 2020; Aquino et al. 2022; Bisht et al. 2021; Misra et al. 2022; Orooji et al. 2021; Özmen et al. 2021; Pishva and Yüce 2021; Sharifi et al. 2021; Sheervalilou et al. 2021; Yadav et al. 2023). By enhancing chemical and biological signals, nanogold particles increase the sensitivity of these nanobiosensors (Thapa et al. 2022). Due to their unique electronic and optical properties, these nanogold particles can detect molecular‐level changes (Khan, Rasmi, et al. 2022). Because coronaviruses can quickly spread among human populations, early detection by nanobiosensors can prevent further spread and thus help control the disease (Ramakrishnan et al. 2021).

Apart from gold nanoparticles, silver nanoparticles are also used in the development of nanobiosensors for diagnosis against viral zoonotic diseases (Samson et al. 2020). It has been reported that silver nanoparticles, in combination with their electrochemical properties, can detect Nipah and Rabies viruses (Gurunathan et al. 2020; Markandan et al. 2022). For instance, research suggests that silver nanoparticles encapsulated with viral enzymes can detect viral RNA in blood or saliva, similar to conventional biosensors (Alhalaili et al. 2020; Ibrahim et al. 2021; Misra et al. 2022; Park et al. 2022). These tools can be important for primary screening, especially in areas where access to advanced laboratories is limited. Besides, owing to their antiviral properties, silver nanoparticles may serve as an active component in multifunctional nanobiosensor systems and as an antimicrobial agent for detection (Aquino et al. 2022; Rahimpour et al. 2021; Yuwen et al. 2023).

Carbon nanotubes are another popular nanomaterial for the fabrication of nanobiosensors. These nanotubes recognize influenza and rabies viruses through their electrical conductivity, stability, and their superior surface area, which is compatible with biological molecules (Arshad et al. 2022; Kaya et al. 2020; Oruganti and Ankireddy 2024; Pirzada and Altintas 2022). The antigens of viruses and antibodies can be detected using carbon nanotubes as sensing substrates. Using carbon nanotube‐based nanobiosensors, influenza virus antigens were detected promptly and precisely directly from respiratory samples (Bardhan et al. 2021; Ehtesabi 2020; Hassanpour et al. 2018). The signals from these nanobiosensors can be easily detected by measuring electrical changes resulting from antigen binding to the surface of carbon nanotubes (Meskher et al. 2023; Ovais et al. 2022). These tools are helpful in detecting pandemic influenza and will help prevent large outbreaks.

Among metal nanoparticles and carbon nanotubes, graphene has also been considered one of the most extensively used nanomaterials in the development of nanobiosensors for the detection of zoonotic viruses (Kim et al. 2021). Because of its two‐dimensional structure and unique electrical properties, graphene effectively detects viruses such as Zika and Chikungunya (Khristunova et al. 2020; Shahrtash et al. 2024). Graphene‐based nanobiosensors can detect molecular changes in samples rapidly and provide sensitive, accurate signals. For example, nanobiosensors based on graphene for the detection of Zika virus RNA can identify viruses even at very low concentrations (Vermisoglou et al. 2020). This feature is of great importance in regions where the Zika virus strikes rapidly, and rapid detection is required.

On the other hand, silica nanoparticles, because of their large surface area and ability to bind biological molecules, were used as substrates for virus detection. These nanoparticles were mainly used to detect viruses, such as the rabies virus (Muttaqien et al. 2022). The silica nanoparticles could be coated with specialized biological coatings that enabled the virus to attach to their surfaces. Nanobiosensors can detect the presence of viruses on surfaces by detecting optical or electrochemical signals generated during attachment (Saylan et al. 2019). Its simplicity and high sensitivity make it suitable for use in a wide variety of settings, including laboratories and rural areas, where precise, rapid diagnosis is required.

Metal oxide nanoparticles with magnetic and electrical properties have been used in the development of a device capable of detecting zoonotic viruses (Ukhurebor et al. 2022). Because of the unique electrical and magnetic properties of the nanoparticles, sensors for rapid detection of viruses, including yellow fever and nipah, can be designed to integrate them (Guliy et al. 2023; Markandan et al. 2022). Iron oxide nanoparticles exhibit a remarkable capacity to generate rapid signals in nanobiosensors that detect magnetic fluctuations caused by viruses (Gambhir et al. 2022; Rezvani Jalal et al. 2021; Wu et al. 2020). Responding promptly and detecting zoonotic viruses that propagate rapidly are particularly valuable for rapidly spreading viruses (Colino et al. 2018).

Other types of nanobiosensors, including those with optical properties, have been developed for the detection of viruses like yellow fever and Zika (Maddali et al. 2021). These nanobiosensors detect optical changes resulting from the binding of viral molecules to nanomaterials and can detect small changes in reflected or transmitted light. Optical nanobiosensors are highly efficient at detecting zoonotic viruses that spread rapidly, thanks to their high sensitivity and versatility across different environments (Sharma et al. 2021).

### 5.3 Parasitic Infections

The transmission of zoonotic parasites from animals to humans is complex and challenging (Esch and Petersen 2013; Pisarski 2019). Mostly caused by parasites that live in human and animal bodies, these diseases can be transmitted to humans through a variety of routes, including water, food, soil, and aerosols, as well as through direct contact with infected animals (Dixon 2021; Hailu et al. 2020; Kakakhel et al. 2021). For effective control and prevention of these diseases, quick and accurate diagnosis is essential. Furthermore, nanobiosensors belong to an advanced technology with extremely high sensitivity and accuracy, which are especially useful for the early diagnosis of diseases like these (Barbosa et al. 2021). *Plasmodium* spp. causes malaria, one of the most widespread parasitic zoonotic diseases. The parasite infects humans through the bite of an infected mosquito (Kojom Foko et al. 2023; Lempang et al. 2022; Sato 2021). The construction of nanobiosensors for the detection of Plasmodium antigens has been undertaken in several studies (Baptista et al. 2022; Dutta 2020; Krampa et al. 2020; Kumar, Singh, et al. 2023; Ukhurebor et al. 2022). The use of gold nanoparticle‐based optical nanobiosensors enables the assessment of optical changes resulting from the binding of malaria antigens to the nanoparticle surface, thereby enabling the identification of parasites in blood (Feyziazar et al. 2022; Patel et al. 2023). Gold nanoparticles enhance optical signals in these nanobiosensors, enabling them to detect parasites at low levels (Debnath and Das 2020).

As one of the most prevalent zoonotic parasitic diseases associated with *Toxoplasma gondii*, toxoplasmosis has great importance (Abdul Hafeez et al. 2022). It has been demonstrated that raw or undercooked meat, as well as contact with cat feces, are capable of spreading the parasite (Almeria and Dubey 2021; Hasan et al. 2024; Sazmand et al. 2019; Soroushianfar et al. 2024). Symptoms of the disease must be diagnosed as quickly as possible in pregnant women to prevent vertical transmission. By utilizing gold and silver nanoparticles, electrochemical nanobiosensors have demonstrated effective results in screening for Toxoplasma antigens in blood as well as other body fluids (Król et al. 2023; Mukherjee and Mukherjee 2021; Rather et al. 2024). By using gold nanoparticles, these nanobiosensors increase detection sensitivity and precision and drastically reduce detection time (Safarpour et al. 2021).

Another important zoonotic parasitic disease is fascioliasis caused by the *Fasciola* spp. (Siles‐Lucas et al. 2021). Fascioliasis is more widespread in areas where contaminated water and food with parasite eggs are consumed (Cwiklinski and Dalton 2022; Kumar et al. 2020; Thakur 2024). Fasciola antigens in blood samples can be successfully and promptly detected by electrochemical and optical nanobiosensors based on carbon nanotubes and gold nanoparticles (Flores‐Ramírez et al. 2024; Kulkarni et al. 2022). With their large surface area and unique structure, carbon nanotubes can bind antigens efficiently and amplify electrical or optical signals. This feature enables early detection of this parasite during the infection stage, allowing timely treatment.

Hydatid cyst caused by *Echinococcus* is another parasitic zoonotic disease, generally transmitted via contact with infected dogs and/or contaminated food and water (Gessese 2020; Pal et al. 2022; Soleymani et al. 2024). In this disease, hydatid cysts form in various body organs, such as the liver and lungs, which can cause serious health problems (Dana et al. 2021; Khan et al. 2020; Mathivathani et al. 2023; Tamarozzi et al. 2020). Magnetic nanobiosensors are an efficient method for the rapid diagnosis of this disease, using magnetic nanoparticles to isolate *E. granulosus* antigens from biological samples (Jafari et al. 2022; Welearegay et al. 2019). Due to their magnetic properties, magnetic nanoparticles can accurately isolate parasite antigens from samples and produce diagnostic signals and electrochemical nanobiosensors.

Another zoonotic parasitic disease, widespread in areas with low sanitation, is giardiasis, caused by the parasite *Giardia duodenalis* (Ayana 2023; Bahramdoost et al. 2021; Morsy et al. 2023). Evidence suggests that contaminated food and water can transmit the parasite and induce serious gastrointestinal complications (Abbas et al. 2022; Bilgiç et al. 2020; Kiani‐Salmi et al. 2019; Moratal et al. 2020). Using optical and electrochemical nanobiosensors, silver and graphene nanoparticles have been shown to rapidly and effectively detect *Giardia* (Ajayi et al. 2022; Feyziazar et al. 2022; Kumar et al. 2024; Nemati et al. 2023; Soumya et al. 2024). Ag silver nanoparticles have been incorporated into nanobiosensors for *Giardia* due to their antibacterial properties and high signaling sensitivity (Çaktü Güler et al. 2024; Ngashangva et al. 2022; Sadanandan et al. 2023). Silver nanobiosensors detect the presence of a parasite at very low concentrations, even in water and fecal samples.

Regarding *Leishmaniasis*, *Leishmania* spp. is transmitted through the bites of infected phlebotomine sand flies (Ahmad et al. 2022; Cecílio et al. 2022; Kumosani et al. 2022; Sadr, Sharifi, et al. 2025; Salah et al. 2020). *Leishmania* can further cause serious skin or visceral ulcers. Various nanobiosensors, including optical and metallic ones, detect parasites' antigens in biological samples with high sensitivity by monitoring changes in the binding of *Leishmania* antigens to the surfaces of nanoparticles (Farooq and Zezell 2024; Feyziazar et al. 2022; Król et al. 2023). Detecting leishmaniasis in its early stages can minimize the spread of the disease in high‐leishmaniasis‐burden areas with such sensors.

Trichinellosis is another parasitic zoonotic disease that is transmitted to humans primarily by eating infected meat raw, especially pork, boar, or other wild game, and is caused by *Trichinella spiralis* (Antolová et al. 2020; Diaz et al. 2020; Pozio and Gomez Morales 2023; Zhang, Wang, and Cui 2022). Infection with this parasite causes serious muscle infections resulting in inflammation and muscle pain (myalgia) and many other problems (Crisóstomo‐Jorquera and Landaeta‐Aqueveque 2022). Electrochemical and optical nanobiosensors based on carbon nanotubes and magnetic nanoparticles have been effectively used for the rapid detection of this parasite in biological samples such as muscle tissue and blood (Gattani et al. 2023). Magnetic nanoparticles are capable of separating parasites out of complex samples, either by producing electrochemical or visible signals. In this process, gold or silver nanoparticles attach to the parasites. A wide range of nanobiosensors, including gold nanoparticles, silver nanoparticles, graphene, carbon nanotubes, and magnetic nanoparticles, are under development for parasitic zoonotic diseases diagnosis. These sensors, especially in areas with high prevalence, will be particularly useful for reducing infection rates and preventing the spread of these parasites, thanks to their high accuracy and sensitivity for fast detection.

## 6 Combining Machine Learning and Nanotechnology

**FIGURE 6:**
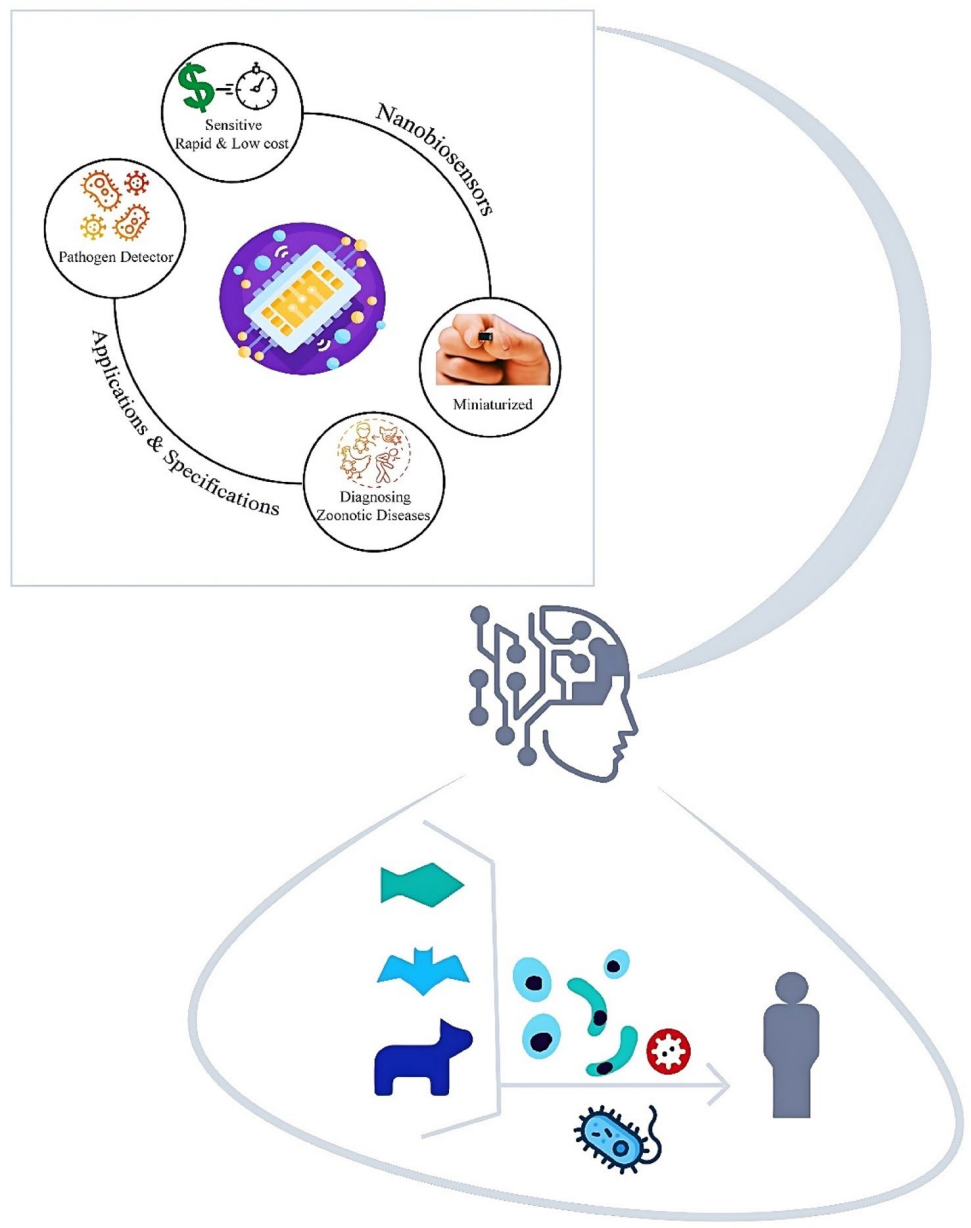
This figure defines the applications and specifications of nanobiosensors that can help gather data for the prediction of zoonotic diseases by artificial intelligence (AI) more easily. Some of these characteristics can be observed, such as pathogen detection, miniaturization, sensitivity, rapidity, and low cost.

Diagnostics and control of these diseases have become increasingly important for public health and the economy. Due to the advent of cutting‐edge technologies, including nanotechnology and machine learning, it is now possible to detect zoonotic diseases more effectively and reliably (Figure 6) (Neethirajan 2017; Wang et al. 2023).

### 6.1 Using Machine Learning Algorithms to Analyze Diagnostic Data

Diagnostic methods using different methods for diagnosis result in a huge amount of data that makes the diagnosis of zoonotic diseases difficult to perform effectively. Machine learning algorithms can significantly enhance the processing and analysis of large amounts of data. These algorithms identify complex patterns in data and thus provide accurate diagnoses of diseases. With the help of classification algorithms such as artificial neural networks and SVMs, nanobiosensor data can be analyzed, and diseases diagnosed (Amethiya et al. 2022; Banerjee et al. 2021; Rahmani et al. 2023). Machine learning offers two benefits for diagnosing zoonotic diseases, namely, prediction and early recognition. The early stages of the disease can be detected through deep learning algorithms, which will help doctors and researchers intervene earlier and more effectively (Ramalingam et al. 2023; Verma et al. 2022). The management of rapidly spreading zoonotic diseases requires this ability. Through the collaboration of nanotechnology and ML, diagnostic processes can be optimized, enhancing speed and accuracy. Machine learning algorithms can be used to interpret nanobiosensor data and identify disease‐related patterns; however, these algorithms require highly accurate and sensitive sensors to determine infection status (Dave et al. 2022). These algorithms identify patterns in nanobiosensor data using analytical techniques and provide accurate diagnostic results. The integration of these two technologies can have potential applications, especially in challenging, complex environments such as rural areas or developing countries, where a scarcity of high‐end medical equipment is common. Portable nanobiosensors integrated with ML algorithms can be distributed in the area for quick, cost‐effective diagnostics (Kokabi et al. 2023). Diagnosis and prevention or reduction in the spread of zoonotic diseases are possible with this approach.

### 6.2 Nanoparticles and Multiple Data Analysis

The unique properties of nanoparticles, in fact, allow them to play an important role in the detection of zoonotic diseases. It is possible to detect several pathogenic agents simultaneously using nanoparticles (Arellano Vidal and Govan 2024). Different nanoparticles can be used together in combination with ML algorithms. It is possible to identify proteins and biomarkers simultaneously using both gold and silver nanoparticles, then analyze the data with ML algorithms to characterize the proteins and biomarkers (Dixon et al. 2023; Enginler et al. 2024).

The quenching of fluorescence by gold and silver nanoparticles can be precisely modeled using modified nanometal surface energy transfer (NSET) equations integrated with ML. By optimizing parameters such as the metal damping constant and orientation factor, and combining experimental data from both types of nanoparticles with models such as a multilayer perceptron and Lasso regression, high *R* ^2^ values (> 0.97) were obtained (Demers et al. 2024). This emphasizes that combining datasets from metal nanoparticles within a physics‐informed ML approach increases predictive precision and facilitates better nanobiosensor design (Demers et al. 2024; Xu et al. 2023).

Such a combined approach allows doctors and researchers to diagnose several zoonotic diseases with a single, simple, quick test and to provide appropriate treatment after analyzing the results (Jia et al. 2020; Moosazadeh et al. 2022; Payedimarri et al. 2021; Yedinak et al. 2021). This application is critical in emergency situations where a quick and accurate diagnosis is critical (Cui et al. 2020; Pan et al. 2024).

### 6.3 Predicting the Spread of Zoonotic Diseases Using Machine Learning

Another important application of ML is in diagnosing diseases and preventing the spread of zoonotic diseases. Predictive ML algorithms can analyze environmental, social, and health data to identify high‐risk areas and prevent epidemics (Alfred and Obit 2021; Devarakonda et al. 2022; Razavi‐Termeh et al. 2021; Zhang, Zhang, et al. 2022). For instance, regression algorithms can predict the probability of disease outbreaks, such as malaria or leishmaniasis, by examining climatic and biological data. The algorithm will enable the health authority to provide the necessary resources and equipment promptly and to take measures to prevent it (Ajagbe and Adigun 2024; Cao et al. 2022; Dogheim and Hussain 2023; Marcus et al. 2020). This method will be able to save thousands of people in the zoonotic disease outbreak‐prone areas due to the reduced cost of the treatment (Dong et al. 2021; Gong et al. 2021; Lăzăroiu et al. 2024; Malki et al. 2020; Mhlanga 2022; Pourghasemi et al. 2020).

### 6.4 Application of Deep Learning in Medical Image Recognition

**FIGURE 7:**
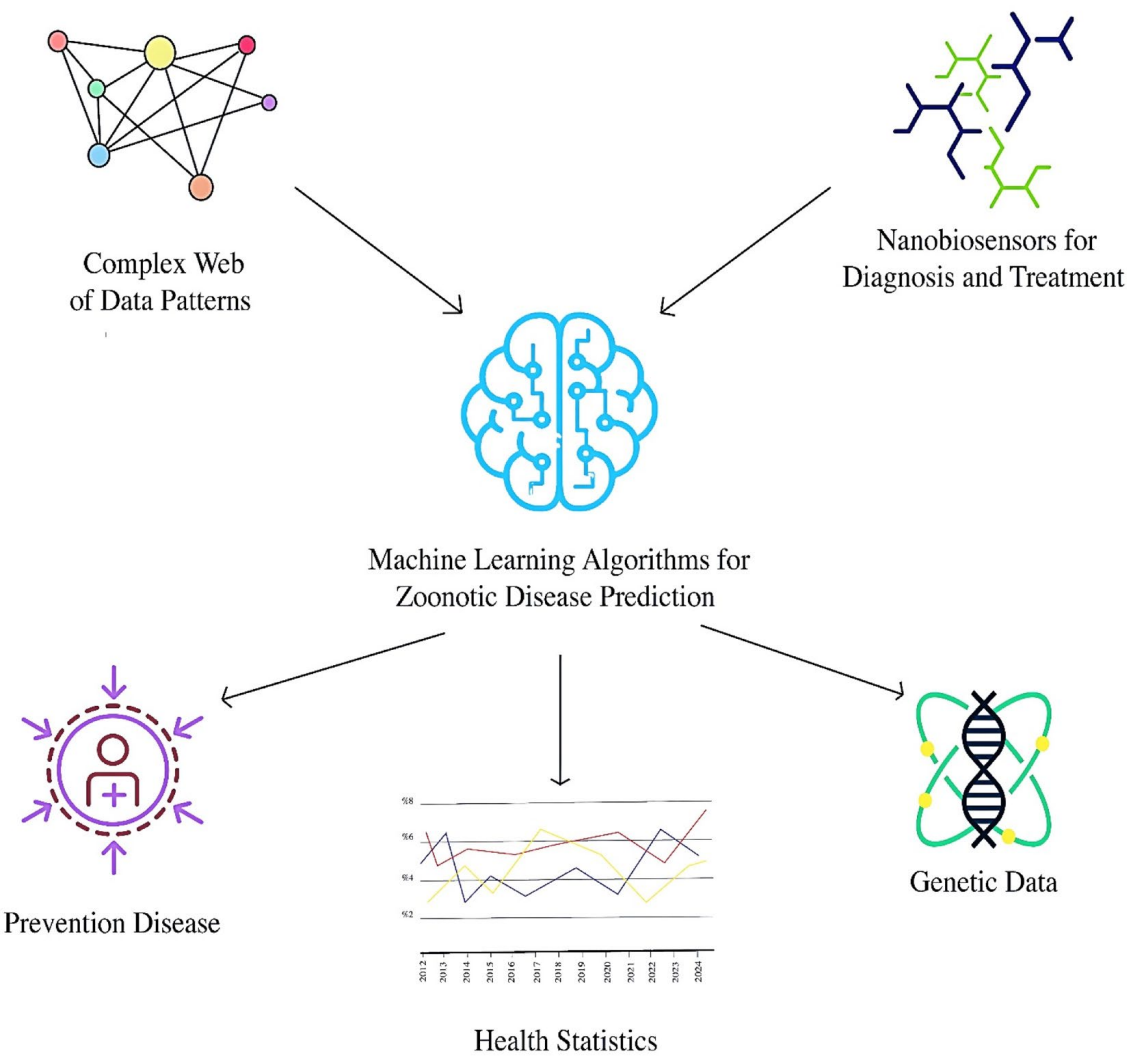
This diagram shows that Machine Learning can gather data from nanobiosensors and complex web patterns using its algorithms to predict, diagnose, and treat zoonotic diseases, and based on these datasets, ML can provide more personalized care and improve health measures.

For diagnosing zoonotic diseases, deep learning algorithms are used alongside ML algorithms (Bhattacharya et al. 2021; da Silva Neto et al. 2022). The algorithms can analyze medical images, such as radiology and magnetic resonance imaging (MRI), and identify symptoms of zoonotic diseases with high precision. By analyzing medical images, Convolutional Neural Networks (CNNs) are particularly effective at diagnosing diseases faster and more accurately. Combining deep learning with nanobiosensors can also help develop automated (Ali 2023; Hu et al. 2022; Ikerionwu et al. 2022). For example, by using images of nanostructures and analyzing them with deep learning algorithms, it is possible to detect the exact state of infection (Figure 7).

## 7 Challenges and Future Prospects

Incorporating ML with nanotechnology raises various issues. Data limitations are considerable, especially since ML requires high‐quality, standardized data, but medical information is often inaccessible, inconsistent, and fragmented. Ensuring data accuracy and consistency is key to the efficiency of ML algorithms. Since ML applications involve sensitive health information, they pose ethical and legal challenges. An important way to ensure regulatory compliance is to improve anonymization and encryption techniques. The technological challenges of nanotechnology also pose a barrier, as producing consistently high‐quality nanoparticles is complex and can be influenced by particle size or structure. Costs and infrastructure constraints would hinder the development and implementation of more advanced and modern technologies in less developed countries. In the absence of a long‐term evaluation of nanotechnology's environmental impact, it is essential to implement strict safety measures and assess the technology's sustainability going forward.

Despite these challenges, the future of ML and nanotechnology in diagnostics looks bright. Portable diagnostic devices incorporating nanobiosensors and ML algorithms will provide quick, affordable diagnoses in resource‐poor settings. In future developments, smart nanoparticles will be able to monitor, detect, and treat diseases in real time with unprecedented precision. Integrating ML into data analysis from nanotechnology‐based field sensors can enhance the ability to detect events and respond to zoonotic diseases. In addition, strong multi‐sectoral collaboration among government agencies, academia, and industry will ensure equitable, innovative development. This, therefore, calls for international standardization on the use of ML applications and nanotechnology, ensuring safety, efficacy, and adherence to ethical considerations that would allow such technologies to realize their full potential to address zoonotic diseases.

## 8 Conclusion

The amalgamation of ML with nanotechnology has exceptional prospects for the identification and treatment of zoonotic diseases. The advantages of these technologies include greater economic efficiency, diagnostic accuracy, and adaptability, in particular in environments with limited resources. Nevertheless, multidisciplinary research and international cooperation will be necessary to overcome obstacles, such as data constraints and ethical dilemmas. Innovative and accessible health systems will be enhanced as a result of this collaboration, thereby reducing the risks associated with zoonotic diseases.

## Author Contributions

**Narges Lotfalizadeh:** writing – original draft (equal), writing – review and editing (equal). **Cinzia Santucciu:** writing – original draft (equal), writing – review and editing (equal). **Valentina Chisu:** writing – original draft (equal), writing – review and editing (equal). **Helia Sepahvand:** writing – original draft (equal), writing – review and editing (equal). **Abbas Rahdar:** conceptualization (lead), methodology (lead), supervision (lead), writing – original draft (equal), writing – review and editing (equal). **Razieh Behzadmehr:** writing – original draft (equal), writing – review and editing (equal). **Octavio Luiz Franco:** writing – original draft (equal), writing – review and editing (equal). **Guettari Moez:** writing – original draft (equal), writing – review and editing (equal). **Luiz Fernando Romanholo Ferreira:** writing – original draft (equal), writing – review and editing (supporting).

## Funding

This work was supported by Conselho Nacional de Desenvolvimento Científico e Tecnológico and Coordenação de Aperfeiçoamento de Pessoal de Nível Superior.

## Consent

The authors have nothing to report.

## Conflicts of Interest

The authors declare no conflicts of interest.

## Related WIREs Articles

Machine Learning and Artificial Intelligence in Nanomedicine

## Data Availability

Data sharing is not applicable to this article as no new data were created or analyzed in this study.
